# Vidofludimus inhibits porcine reproductive and respiratory syndrome virus infection by targeting dihydroorotate dehydrogenase

**DOI:** 10.1186/s13567-023-01251-0

**Published:** 2023-12-20

**Authors:** Yuanqi Yang, Yanni Gao, Lujie Zhang, Xing Liu, Yangyang Sun, Juan Bai, Ping Jiang

**Affiliations:** 1 Key Laboratory of Animal Diseases Diagnostic and Immunology, Ministry of Agriculture, MOE International Joint Collaborative Research Laboratory for Animal Health & Food Safety, College of Veterinary Medicine, Nanjing Agricultural University, Nanjing, 210095 China; 2 Jiangsu Co-innovation Center for the Prevention and Control of Important Animal Infectious Diseases and Zoonoses, Yangzhou University, Yangzhou, 225009 China

**Keywords:** Vidofludimus, PRRSV infection, antiviral, dihydroorotate dehydrogenase, broad-spectrum

## Abstract

Porcine reproductive and respiratory syndrome virus (PRRSV) infection has caused huge economic losses in global swine industry over the last 37 years. PRRSV commercial vaccines are not effective against all epidemic PRRSV strains. In this study we performed a high-throughput screening (HTS) of an FDA-approved drug library, which contained 2339 compounds, and found vidofludimus (Vi) could significantly inhibits PRRSV replication in Marc-145 cells and primary porcine alveolar macrophages (PAMs). Compounds target prediction, molecular docking analysis, and target protein interference assay showed that Vi interacts with dihydroorotate dehydrogenase (DHODH), a rate-limiting enzyme in the de novo pyrimidine synthesis pathway. Furthermore, PRRSV infection was restored in the presence of excess uridine and cytidine which promote pyrimidine salvage, or excess orotate which is the product of DHODH in the de novo pyrimidine biosynthesis pathway, thus confirming that the antiviral effect of Vi against PRRSV relies on the inhibition of DHODH. In addition, Vi also has antiviral activity against Seneca virus A (SVA), encephalomyocarditis virus (EMCV), porcine epidemic diarrhea virus (PEDV), and pseudorabies virus (PRV) in vitro. These findings should be helpful for developing a novel prophylactic and therapeutic strategy against PRRSV and other swine viral infections.

**Supplementary Information:**

The online version contains supplementary material available at 10.1186/s13567-023-01251-0.

## Introduction

Porcine reproductive and respiratory syndrome (PRRS), also known as porcine blue ear disease, is a highly infectious swine disease worldwide caused by porcine reproductive and respiratory syndrome virus (PRRSV) and characterized by reproductive failure in sows and respiratory diseases in all pigs [1–3]. PRRSV infections could be divided into subclinical, lethal, and persistent infections in terms of the pig growth stage and immune status, the virus strains and secondary or co-infected pathogens, environmental conditions, and disease management level [4, 5]. PRRSV is a small enveloped, single-stranded positive-sense RNA virus belonging to the genus *Porartevirus*, family *Arteriviridae*, and order *Nidovirales* [6]. Currently, PRRSV can be classified into two genotypes, i.e., the European genotype and the North American genotype [7]. High genetic diversity is a significant characteristic of PRRSV. PRRSV mutates rapidly at an estimated rate of 3.29 × 10^–3^ substitutions per nucleotide site per year, developing growing evolutionary strains [8–11]. The PRRSV strains prevalent in China are mainly the North American genotype.

Nowadays, vaccination is the most common strategy for PRRS prevention and control. Different kinds of vaccines are now in market, such as inactivated vaccine [12], modified-live virus (MLV) vaccine [13], recombinant vector vaccine [14], DNA vaccine [15], and subunit vaccine [16]. However, these commercially available vaccines can provide only incomplete protections [17–19]. Therefore, more effective and safe methods for PRRSV control are urgently in need.

Antiviral drugs occupy an important role in disease prevention and control. Natural compounds and compositions provide valuable sources for antiviral drugs, many of which have shown anti-PRRSV activity in vitro, such as proanthocyanidin A2 [20], griffithsin [21], (-)-epigallocatechin-3-gallate [22], tea polyphenols [23], and Emodin [24]. However, so far there is still no commercial antivirals against PRRSV.

In this study, we screened a library of 2339 FDA-approved drugs and firstly identified four new-hit compounds as potential antivirals for PRRSV treatment after three rounds of screening. Among them, vidofludimus significantly inhibited PRRSV infection with the highest select index (SI) by directly targeting dihydroorotate dehydrogenase (DHODH), and showed a general antiviral activity against other swine viruses, demonstrating excellent potential as a broad-spectrum antiviral product.

## Materials and methods

### Cells, viruses, and reagents

Marc-145 cells (an African green embryonic kidney epithelial cell line, ATCC) were cultured in Dulbecco’s modified Eagle’s medium (DMEM; Invitrogen, USA) supplemented with 10% fetal bovine serum (FBS; Gibco, USA) and Penicillin (250 U/mL)-Streptomycin (250 μg/mL) at 37 °C in a humidified atmosphere containing 5% CO_2_. Porcine alveolar macrophages (PAMs) were collected from lung lavages of 6-week-old Yorkshire pigs (free of PRRSV, PCV2, PRV), as previously described [11], and cultured in Roswell Park Memorial Institute 1640 medium (RPMI 1640; Gibco, USA) containing 10% FBS and Penicillin (250 U/mL)-Streptomycin (250 μg/mL) at 37 °C in a humidified atmosphere containing 5% CO_2_. Three North American genotype PRRSV strains were employed. The highly pathogenic PRRSV strain BB0907 (GenBank accession no. HQ315835.1) was used for all experiments and represented as “PRRSV” in this article unless otherwise specified. The PRRSV strains S1 (a classical strain; GenBank accession no. DQ459471.1) and FJ1402 (a NADC30-like strain; GenBank accession no. KX169191.1) were used and named as S1 and FJ1402, respectively. These PRRSV strains were all maintained in our laboratory. PEDV YZ (GenBank accession no. MK841495.1), PRV ZJ01 (GenBank accession no. KM061380.1), SVA CH-SD (GenBank accession no. MH779611.1), and EMCV NJ08 (GenBank accession no. HM641897) were maintained in laboratory. Vidofludimus (Vi), dihydroorotate (DHO), orotate (ORO), uridine, and cytidine were purchased from Selleck Chemicals (purity > 99%; Selleck Chemicals, USA). 6-azauracil (6-AU), an inhibitor of orotidine 5'-phosphate decarboxylase (ODCase), was purchased from Sigma (purity > 98%; Sigma-Aldrich, USA).

### Screening of a natural product library

**Figure 1:**
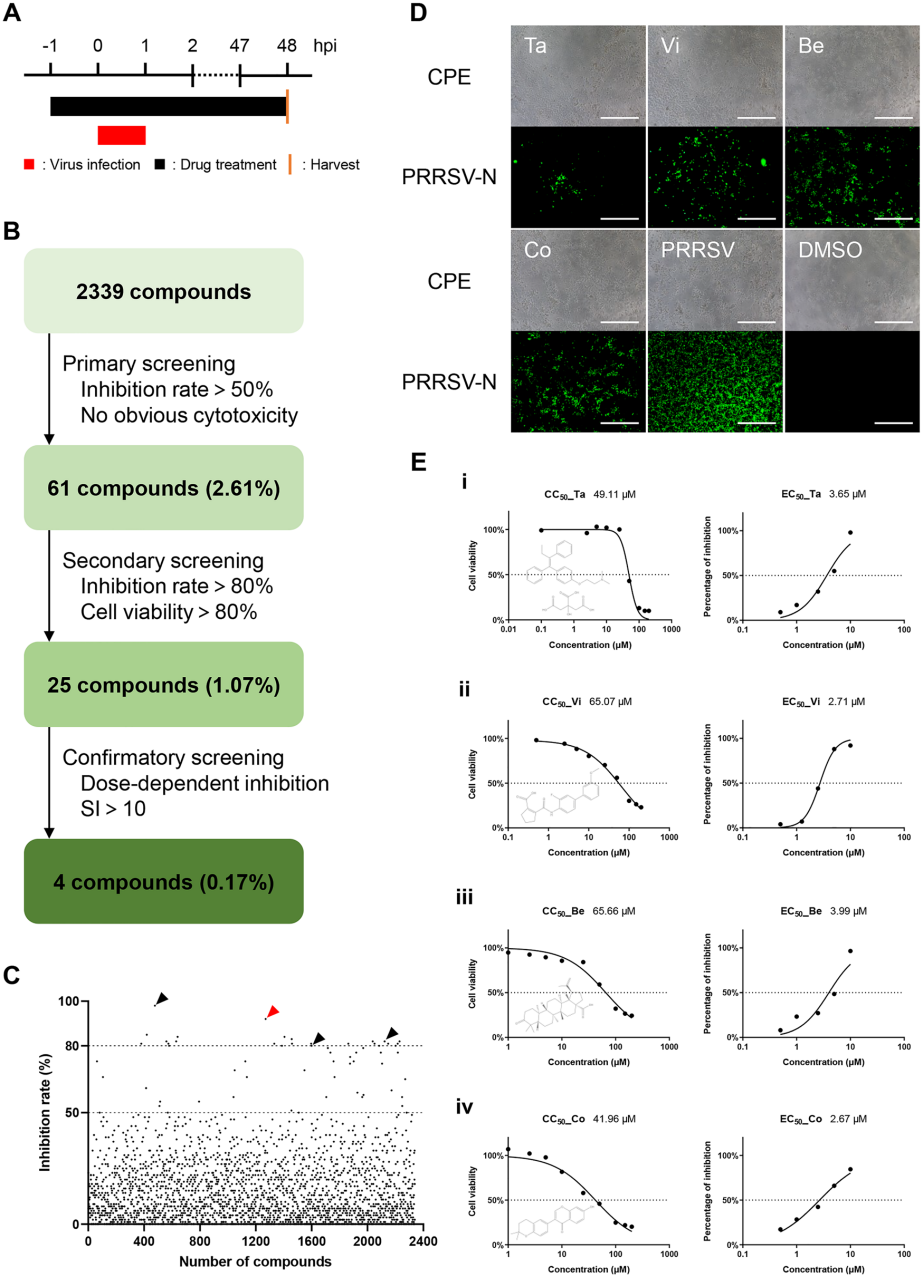
**High-throughput screening (HTS) for inhibitors of PRRSV infection from an FDA-approved drug library. A** HTS assay timeline. **B** HTS assay flowchart. **C** Each dot represents the percent inhibition of PRRSV (0.01 MOI) achieved with by compound (5 μM). **D** IFA of infected Marc-145 cells treated with one of the four designated compounds. PRRSV N protein is colored green, and brightfield-imaged cells show CPE. Scale bars, 500 μm. **E** CC_50_ and EC_50_ of Ta (i), Vi (ii), Be (iii) and Co (iv). Insets show the structure of each compound.

An FDA-approved library containing 2339 compounds was purchased from Selleck Chemicals and stored as 10 mM stock solutions in DMSO at -80 °C until use. The workflow for high-throughput screening (HTS) was carried out as shown in Figures 1A and B. Marc-145 cells were infected with PRRSV (0.01 multiplicity of infection (MOI)) or mock-infected. Drug treatment (5 µM compound or DMSO) was performed from 1 h before virus infection until cells were collected for cytopathic effect (CPE) observation and indirect immunofluorescence assay (IFA) analysis at 48 h post-infection (hpi). Fluorescence intensity was measured by ImageJ software. The inhibition rate of each compound was normalized to the equal volume of DMSO control group. Each assay was performed in duplicate.

During the primary screening, compounds were weed out if they showed any observable cytotoxicity or demonstrated a less than 50% reduction of CPE compared to the DMSO control group. For the second round of screening, compounds displaying an over 80% of cell viability and the inhibition rate of PRRSV infection were selected. In the final round of screening, 50% effective concentration (EC_50_) and 50% cytotoxic concentration (CC_50_) of each remaining candidate compound were calculated using the log (inhibitor) vs. response—Variable slope (four parameters) method by GraphPad Prism 7.0 software (GraphPad Software; CA, USA). The compounds which displayed a dose-dependent inhibition activity on PRRSV infectivity and a selectivity index (SI) higher than ten were considered for further study.

### Cell viability assay

Marc-145 cells were treated with compounds or transfected with siRNAs, and incubated for 48 h at 37 °C in a humidified atmosphere containing 5% CO_2_. Cell viability was tested using an enhanced Cell Counting Kit-8 (CCK-8; Beyotime, China) following the manufacturer’s instructions. The CC_50_ was calculated using GraphPad Prism 7.0 software. DMSO was used as the negative control.

### PRRSV infectivity inhibition assay

Indirect immunofluorescence assay (IFA) was used to examine the effect of the compounds, including tamoxifen citrate (Ta), vidofludimus (Vi), betulonic acid (Be), and corylin (Co), on PRRSV infectivity. Cells were treated with two-fold serially diluted compounds (1 µM to 10 µM), and infected with PRRSV (0.01 MOI) for 48 h at 37 °C. Cells were fixed with 4% paraformaldehyde for 20 min and permeabilized with 0.1% Triton X-100 for 30 min at 37 ℃. PRRSV infectivity was detected with a mouse anti-PRRSV N-protein mAb (1:200 dilution, made in laboratory [25]) as primary antibody and the Alexa Fluor 488-conjugated goat anti-mouse IgG (H–L) (1:200 dilution; Proteintech, USA) as secondary antibody. Nuclei were stained with DAPI (Beyotime, China) for 10 min at room temperature. Immunofluorescence was observed using a Zeiss inverted fluorescence microscope. Fluorescence intensity was determined by ImageJ software. The EC_50_ of each compound was estimated by GraphPad Prism 7.0 software. The SI was determined by the ratio of CC_50_ to EC_50_.

### Western blot assay

Cells were lysed with 100 μL of Radio-immunoprecipitation assay (RIPA) lysis buffer (Beyotime, China) for 15 min on ice, then resolved by SDS-PAGE and transferred to a nitrocellulose membrane. The membrane was then blocked with 5% non-fat milk in PBST (w/v) and then probed with the following antibodies: anti-PRRSV N-protein mAb (1:1000 dilution), anti-SVA VP2-protein mAb (1:1000 dilution), anti-EMCV VP1-protein mAb (1:1000 dilution), anti-PEDV N-protein mAb (1:1000 dilution), or anti-PRV gB-protein mAb (1:1000 dilution) prepared in our laboratory; and anti-β-actin mAb (1:10 000 dilution; Proteintech, USA), anti-DHODH mAb (1:2000 dilution; Proteintech, USA), or anti-HA mAb (1:5000 dilution; BioWorld, USA) as primary antibody, respectively. Horseradish peroxidase (HRP)-conjugated goat anti-rabbit or goat anti-mouse IgG (H–L) were used as secondary antibodies (1:1000 dilution; Beyotime, China).

### RNA extraction and quantitative real‑time PCR

Total RNA was extracted from cells using a Total RNA Kit I (Omega Bio-Tek, USA). RNA was then reverse transcribed into cDNA using HiScript qRT SuperMix (Vazyme, China) following the manufacturer’s instructions. Quantitative RT-PCR was performed using an ABI QuantStudio 6 Systems (Applied Biosystems, USA) with AceQ^®^ qPCR SYBR^®^ Green Master Mix (Vazyme, China) following the manufacturer’s instructions. Gene quantification was referenced to monkey GAPDH or pig β-actin genes, normalized to the mock-infected control and calculated through 2^−ΔΔCt^ methods and the results were calculated as mean ± standard deviation (SD). The primers are listed in Table 1.

**Table 1:** **Sequences of primers and siRNAs used in the study.**

Primer/siRNA	Sequence (5’-3’)
PRRSV ORF7-F	AAACCAGTCCAGAGGCAAG
PRRSV ORF7-R	TCAGTCGCAAGAGGGAAAT
monkey HPSE-F	CTTCGTACCTTGGCCAGAGG
monkey HPSE-R	CTTCTCCACCAGCCTTCAGG
monkey HSPG2-F	CCTGACGGCCACTTCTACC
monkey HSPG2-R	GCAGGCATCACCACATTCAC
monkey Sdc-4-F	CACTGAAACCAAGAAACTG
monkey Sdc-4-R	GTTAGACACATCCTCACTT
monkey CD163-F	TTCACTGCACTGGGACTGAG
monkey CD163-R	AGGACAGTGTTTGGGACTGG
monkey GAPDH-F	CCTTCCGTGTCCCTACTGCCAA
monkey GAPDH-R	GACGCCTGCTTCACCACCTTCT
pig β-actin-F	CTCCATCATGAAGTGCGACGT
pig β-actin-R	GTGATCTCCTTCTGCATCCTGTC
DHODH-F _ *Eco*RI	CGAATTCATGGCGTGGAGACA
DHODH-R _ *Xho*I	CGCTCGAGTCAAGCGTAATCTGGAACATCGTATGGGTACCTCCGATGATCTGC
siRNA-1-sense	AAGCCGUGGACGGACUUUAUAdTdT
siRNA-1-antisense	UAUAAAGUCCGUCCACGGCUUdTdT
siRNA-2-sense	GGUAUGGAUUUAACAGUCACGdTdT
siRNA-2-antisense	UGACUGUUAAAUCCAUACCUGdTdT
siRNA-3-sense	CGGGAUUUAUCAACUCAAACCdTdT
siRNA-3-antisense	UUUGAGUUGAUAAAUCCCGGAdTdT
siNC-sense	UUCUCCGAACGUGUCACGUdTdT
siNC-antisense	ACGUGACACGUUCGGAGAAdTdT

### Virus titration

Cells were infected with ten-fold serial dilutions of indicated viruses in eight replicates. After 1 h inoculation at 37 °C, the cells were washed and incubated with fresh medium for 2–5 days. Viral titers were determined using endpoint dilution analysis five days post-inoculation (dpi). The cytopathic effect was observed using an inverted microscope. The median tissue culture infectious dose (TCID_50_) was determined by the Reed-Muench method.

### Virucidal activity assay

To evaluate the virucidal activity of Vi, Vi (10 µM) or DMSO was incubated with PRRSV (0.1 and 1 MOI) for 3 h at 37 °C. The mixtures were then subjected to virus titration as described above.

### Virus binding assay

Marc-145 cells or PAMs were pre-chilled for 1 h at 4 °C before treated with Vi (5 or 10 µM) for 1 h at 4 °C. Then the cells were infected with the mixture of PRRSV (1 MOI) and Vi (5 or 10 µM) for 1 h at 4 °C. Cells were washed 3 times with ice-cold PBS before viral RNA were extracted and quantified by qRT-PCR as described above.

### Virus internalization assay

Marc-145 cells pre-treated with cycloheximide (CHX; 10 μg/mL) for 12 h before PRRSV infection (1 MOI) for 1 h at 4 °C to allow virus attachment. Cells were washed 3 times with ice-cold PBS to remove unbound virus, following by 2 h incubation with fresh DMEM containing 10 µM Vi or DMSO at 37 °C. Cells were washed with citrate buffer (pH 3.0) to remove the non-internalized virus and viral RNA were extracted and quantified by qRT-PCR as described above.

### Virus replication assay

Marc-145 cells were infected with PRRSV (1 MOI). At 6 hpi, the cells were washed 3 times with PBS and then incubated with fresh medium containing Vi (10 µM) or DMSO at 37 °C. Viral RNA were extracted and quantified by qRT-PCR at indicated time post infection as described above.

### Virus release assay

Marc-145 cells were infected with PRRSV (0.1 MOI). At 24 hpi, cells were washed 3 times with PBS and incubated with fresh medium containing Vi (10 µM) or DMSO for 10, 30, and 60 min at 37 °C. Cell supernatants were harvested at indicated time points for virus titration as described.

### Effect of DHODH overexpression on PRRSV replication

DHODH gene was amplified with cDNA from Marc-145 cells and cloned into the pCAGGS with an HA tag at its 3’ end to produce pCAGGS-chloDHODH. The primers are listed in Table 1. Marc-145 cells were transfected with 0, 0.1, 0.2, 0.3, or 0.5 μg of pCAGGS-chloDHODH using Lipofectamine™ 3000 (Invitrogen, USA) according to the manufacturer’s recommendations. At 24 h post-transfection (hpt), the cells were infected with PRRSV (0.1 MOI) or treated with Vi (3 μM) and then infected with PRRSV (0.1 MOI). Cells were harvested at 48 hpi for Western blotting detection as described above.

### Effect of DHODH interference on PRRSV replication

Marc-145 cells were transfected with 100 nM of siDHODH (Biotend, China) or negative control (siNC) using Lipofectamine™ 3000 reagent (Invitrogen, USA). At 18 hpt, the cells were treated with Vi and infected with PRRSV (0.4 MOI). Cells were harvested at 36 hpi for Western blotting detection as described above. The sequences of siDHODH were shown in Table 1.

### Target proteins prediction using SwissTargetPrediction

The structure of Vi was analyzed by the PubChem database and put into the SwissTargetPrediction for identification of potential drug targets in *Homo sapiens* [26–28].

### In silico docking

The crystal structure of *rat* DHODH (ratDHODH) was obtained from the Protein Data Bank (PDB: 1UUO). The putative 3D structure of *chlorocebus sabaeus* DHODH (chloDHODH) and *sus scrofa* DHODH (susDHODH) were predicted and scored using the online tool SWISS-MODEL [29]. Quality assessments of the predicted 3D models, including Ramachandran plot score and Z-score, were performed using the online tools SAVES v6.0 and ProSA-web [30–32]. The 3D structure of vidofludimus (Vi) was obtained from PubChem (Compound CID: 9820008).

The Autodock 4.2 program (genetic algorithm) was used for the docking of Vi to chloDHODH. The estimated free energy of binding was ranked, and the top one complex was employed. The docking results were visualized using PyMOL 2.3.2.

### Molecular dynamic simulation

Thermodynamic constancy of the receptor-ligand system was analyzed through the Gromacs2021.2 software [33, 34]. Firstly, AmberTools22 was used to add GAFF force field to the small molecule. Gaussian 16W carried out hydrogenation of small molecules and calculation of RESP potential, and the potential data would be added to the molecular dynamics system topology file. Simulations were conducted with the Gromacs package using Amber99sb-ildn force field at static temperature 300 K and 1 bar pressure. Long-range electrostatic interactions were treated with the particle-mesh Ewald method. The Tip3p water model was used to solvate the protein in a periodic dodecahedron box extending 10 Å from the nearest protein atom. The total charge of the simulation system was balanced by adding an appropriate amount of Na^+^, minimized by the steepest descent method, and equilibrated with isothermal isovolumic ensemble (NVT) and isothermal isobaric ensemble (NPT) for 100 000 steps, respectively, with the coupling constant of 0.1 ps and the duration of 100 ps. All bond lengths were constrained with the LINear Constraint Solver algorithm. A cut-off of 14 Å was used to calculate short-range van der Waals and electrostatic interactions. Finally, the free molecular dynamics simulation was performed. The time step was 2 fs and the total simulation time was 100 ns. The root-mean-square deviation (RMSD) and the number of hydrogen bonds between ligand and active pockets of the proteins were analyzed to judge binding stability and convergence.

### Broad‑spectrum antiviral assessment

Western blot and TCID_50_ were performed to examine the Vi antiviral activity against other swine disease viruses. BHK-21 cells were infected with SVA (0.02 MOI) or EMCV (0.02 MOI), Vero cells were infected with PEDV (0.1 MOI), and PK-15 cells were infected with PRV (0.1 MOI), with the addition of Vi (0–10 μM) in culture medium. The cells and supernatants were harvested at different time points as indicated.

### Statistical analysis

All statistical analyses were performed using GraphPad Prism 7.0 (GraphPad Software, USA). Results are expressed as the mean ± standard deviation (SD). The significance of differences among groups was determined by one-way or two-way analysis of variance (ANOVA). The asterisks indicate significant differences (*P < 0.05; ** P < 0.01; *** P < 0.001; **** P < 0.0001; ns, not significant).

## Results

### Library screening

In order to detect the effect of the compounds on PRRSV infection, Marc-145 cells were treated with 5 µM compounds and infected with PRRSV as illustrated in Figure 1A. After primary screening, 61 (2.61%) compounds showing no apparent cytotoxicity and 50% CPE reduction compared to the DMSO group were found. These 61 compounds were then subjected to a second round of screening and 25 compounds leading to negligible cytotoxicity and over 80% inhibition rate were screened. After a final screening with the 25 compounds, 4 compounds, including tamoxifen citrate (Ta), vidofludimus (Vi), betulonic acid (Be) and corylin (Co), showed PRRSV inhibition activity in a dose-dependent manner and exhibited an SI higher than 10 (Figures 1B–E, Table 2). Vi was selected for further study as it showed a highest SI of 24.01.

**Table 2:** **CC**_**50**_**, EC**_**50**_**, and SI (SI = CC**_**50**_**/EC**_**50**_**) of each compound (determined in Marc-145 cells).**

Hit compounds	CC_50_ (μM)	EC_50_ (μM)	Select index (SI)
Tamoxifen citrate (Ta)	49.11	3.65	13.45
Vidofludimus (Vi)	65.07	2.71	24.01
Betulonic acid (Be)	65.66	3.99	16.46
Corylin (Co)	41.96	2.67	15.72

### Vidofludimus inhibits PRRSV infection

**Figure 2:**
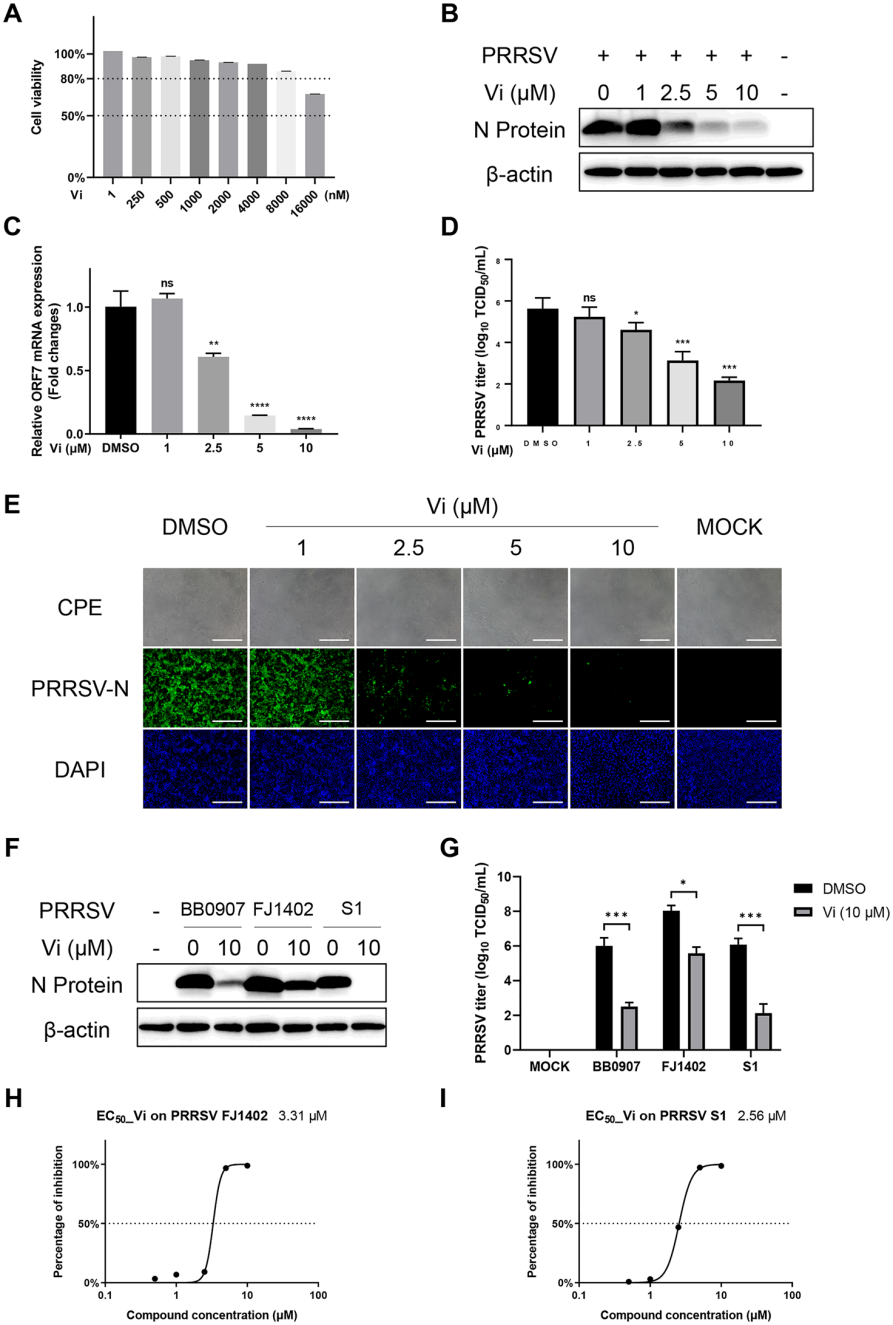
**Vidofludimus anti‑PRRSV activity in Marc‑145 cells. A** Viability of Marc-145 cells treated with the indicated concentrations of Vi for 48 h. **B** Western blot of N-protein in cells infected with PRRSV and treated with the indicated concentrations of Vi. **C** Relative PRRSV ORF7 mRNA levels determined by qRT-PCR. GAPDH was used as reference control. **D** Virus titration by TCID_50_ calculation. **E** Light microscopy and IFA detection of Marc-145 cells (PRRSV-infected and Vi-treated), at 48 hpi. Green, PRRSV N-protein; blue, nucleus. Scale bars, 500 μm. **F** and** G** Western blot of N-protein and TCID_50_ detection in cells infected with different PRRSV genotypes Vi or DMSO treatment. **H** EC_50_ of Vi on PRRSV NADC30-like strain FJ1402. **I** EC_50_ of Vi on PRRSV classical strain S1. Error bars represent the mean ± SD. *, *P* < 0.05; **, *P* < 0.01; ***, *P* < 0.001; ****, *P* < 0.0001; ns, not significant.

The cytotoxicity of Vi was detected on Marc-145 cells with different concentrations of Vi from 1 to 16,000 nM. As shown in Figure 2A, the cell viability of Marc-145 cells was less than 80% until Vi reaches 16 µM. Therefore, the anti-PRRSV activity of Vi were detected with 1, 2.5, 5, and 10 µM. Western blotting of PRRSV N protein, qRT-PCR of ORF7 mRNA, TCID_50_ analysis, CPE, and IFA observation all showed a dose-dependent antiviral activity of Vi against PRRSV (Figures 2B–E). The anti-PRRSV activity of Vi was next confirmed with PRRSV NADC30-like strain FJ1402 and the classical strain S1. The results showed a general antiviral activity of Vi against different PRRSV strains (Figures 2F, G), and the EC_50_ of Vi on PRRSV FJ1402 and PRRSV S1 were 3.31 μM and 2.56 μM, respectively (Figures 2H, I).

**Figure 3:**
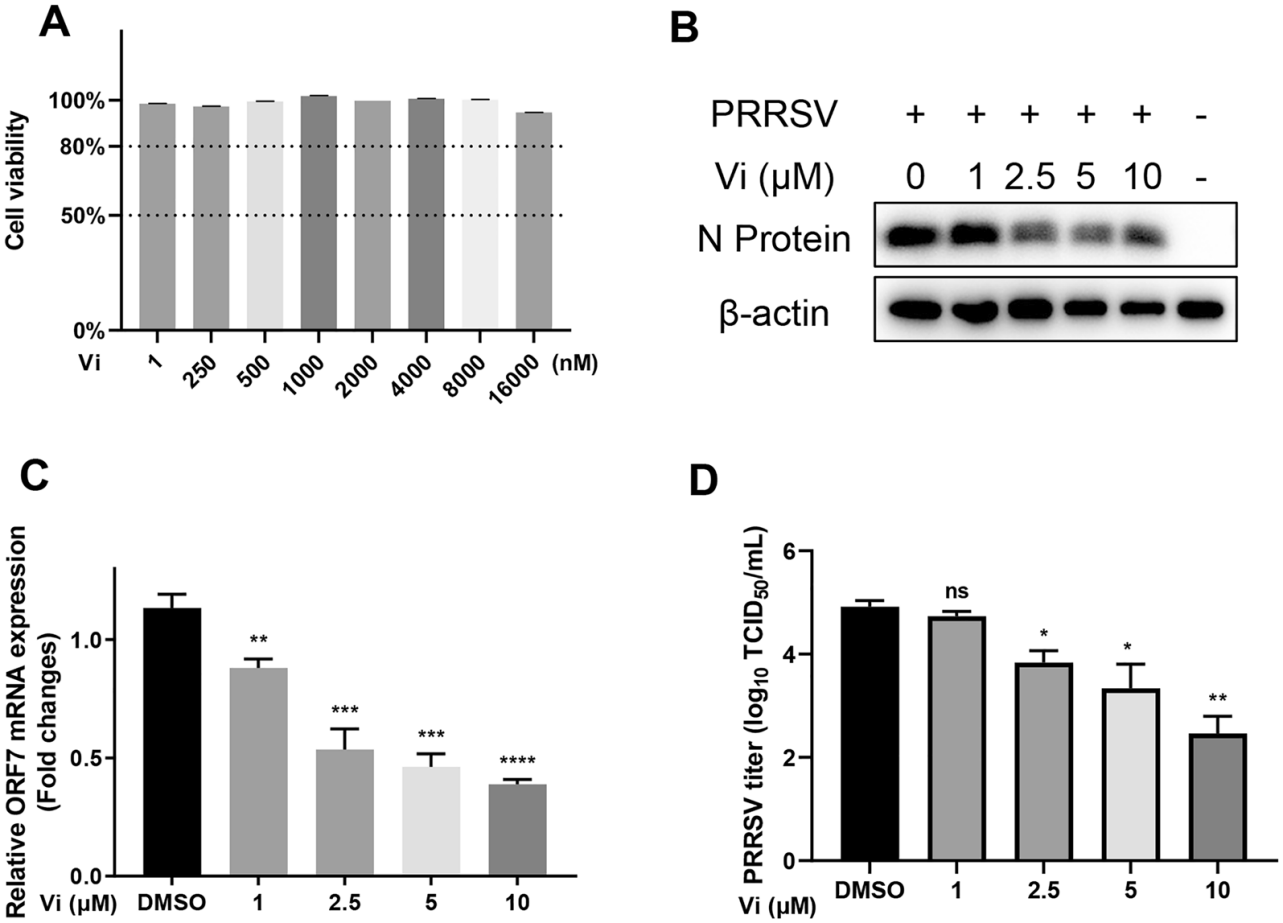
**Vidofludimus anti‑PRRSV activity in PAMs. A** Viability of PAMs treated with the indicated concentrations of Vi for 48 h. **B** Western blot of N-protein in cells infected with PRRSV and treated with the indicated concentrations of Vi. **C** Relative PRRSV ORF7 mRNA levels determined by qRT-PCR. GAPDH was used as reference control. **D** Virus titration by TCID_50_ calculation. Error bars represent the mean ± SD. *, *P* < 0.05; **, *P* < 0.01; ***, *P* < 0.001; ****, *P* < 0.0001; ns: not significant.

The anti-PRRSV activity of Vi was further explored in primary porcine cells, porcine alveolar macrophages (PAMs). Cell viability analysis showed that Vi exhibited little cytotoxicity on PAMs at various concentrations until 16 000 nM (Figure 3A). Western blotting of PRRSV N protein, qRT-PCR of ORF7 mRNA, and TCID_50_ analysis showed a significant and dose-dependent anti-PRRSV activity of Vi in PAMs (Figures 3B–D).

### Vidofludimus inhibits PRRSV infection during virus binding and replication stage

**Figure 4:**
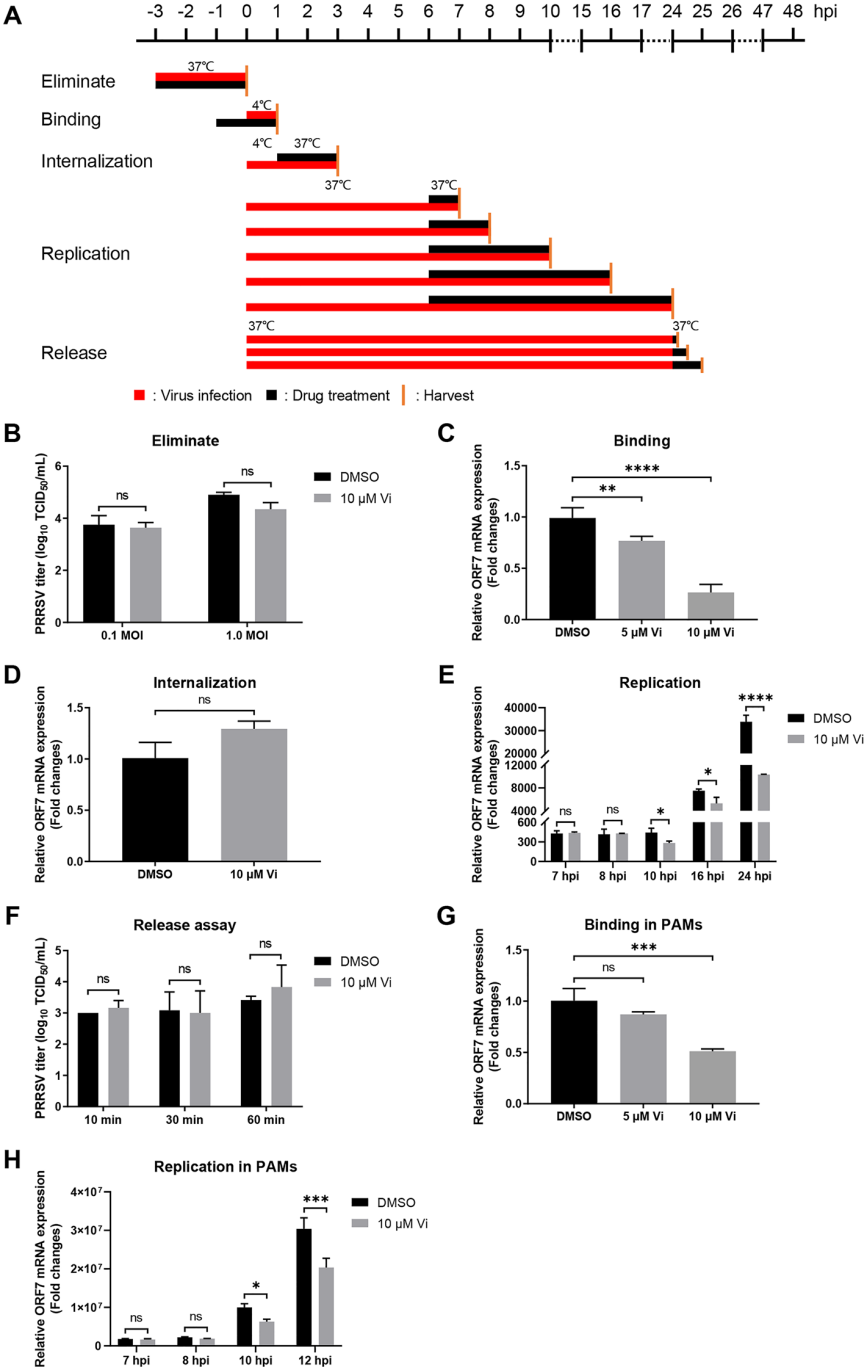
**Time-of-addition analysis of vidofludimus anti-PRRSV activity. A** Schematic illustration of the time-of-addition experiment. DMSO was used as the solvent of Vi. And an equal volume of DMSO was used for treating viruses as black solvent control. **B** TCID_50_ detection for virucidal activity assay. **C** PRRSV ORF7 mRNA levels determination by qRT-PCR in virus binding assay. GAPDH was used as reference control. **D** PRRSV ORF7 mRNA levels determination by qRT-PCR in virus internalization assay. GAPDH was used as reference control. **E** PRRSV ORF7 mRNA levels determination by qRT-PCR in virus replication assay. GAPDH was used as reference control. **F** Virus titration by TCID_50_ calculation with cell supernatant in virus release assay. **G** PRRSV ORF7 mRNA levels determination by qRT-PCR in virus binding assay in PAMs. β-actin was used as reference control. **H** PRRSV ORF7 mRNA levels determination by qRT-PCR in virus replication assay in PAMs. β-actin was used as reference control. Error bars represent the mean ± SD. *, *P* < 0.05; **, *P* < 0.01; ***, *P* < 0.001; ****, *P* < 0.0001; ns: not significant.

Marc-145 cells were infected with PRRSV with Vi treatment at different stages as shown in Figure 4A. The results showed that Vi exhibited no elimination activity against PRRSV in vitro (Figure 4B), and did not affect PRRSV infection during virus internalization and release stage (Figures 4D and F). Interestingly, when Vi was added into the Marc-145 cells during virus binding and replication stage, quantification of PRRSV ORF7 mRNA showed a significantly reduction in the Vi-treatment group compared to the DMSO-treatment group, indicating that Vi inhibited PRRSV infection during virus binding and genome replication stage in Marc-145 cells (Figures 4C, E). Meanwhile, Vi also significantly inhibited PRRSV binding and replication in PAMs cells (Figures 4G, H).

### Vidofludimus stably binds to DHODH in silico

**Figure 5:**
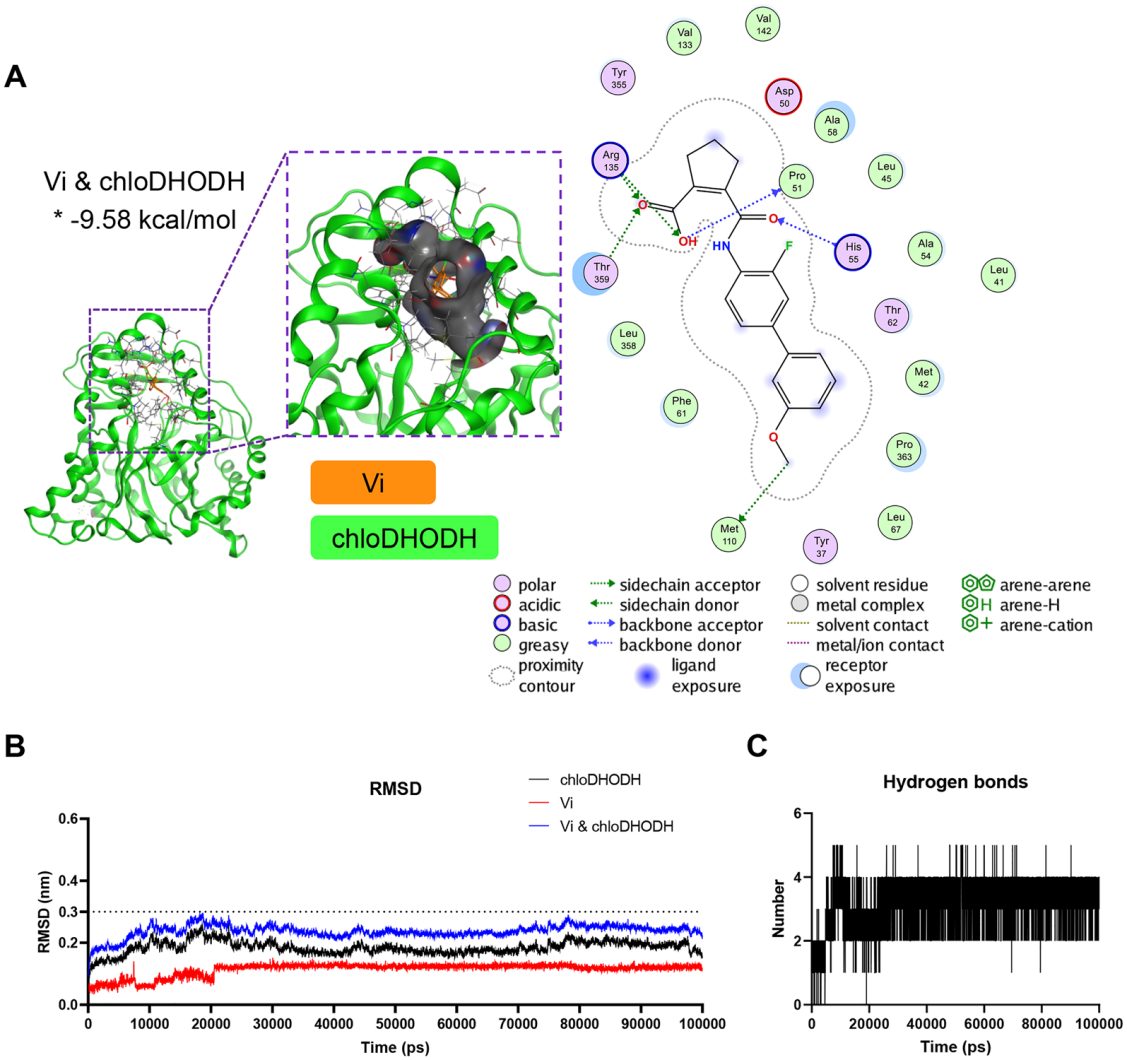
**Target analysis of vidofludimus. A** Docked conformation of chloDHODH with Vi. The compound and protein are represented as sticks and cartoons, respectively. Vi is colored orange and the protein chloDHODH is colored green. The binding site is shown as cavity structure. The binding energy of the Vi-chloDHODH complex is marked with an asterisk. **B** RMSD values of chloDHODH (black), Vi (red), and complex (Vi & chloDHODH, blue) over the 100 ns simulation time. **C** Number of hydrogen bonds involved in the interaction between chloDHODH and Vi during the MD simulation.

In order to explore the molecular mechanism that Vi inhibited PRRSV infection, the target host proteins of Vi was predicted by SwissTargetPrediction. Ten host proteins were exported as possible targets against Vi, of which the oxidoreductase dihydroorotate dehydrogenase (DHODH) showed a probability of 1 (Table 3). The structure of *chlorocebus sabaeus* DHODH (chloDHODH) was predicted using the online tool SWISS-MODEL and the reliability of the predicted structure was analyzed on SAVES v6.0 and ProSA-web. The Ramachandran plot analysis of chloDHODH revealed 94.1, 5.9, 0, and 0% residues in the most favorable, additional allowed, generously allowed, and disallowed regions, respectively (Additional file 1A), and a Z-score value of -9.57 (Additional file 1B). The binding activity between Vi and chloDHODH was analyzed using Autodock. Vi was shown to bind chloDHODH with a binding energy of -9.58 kcal/mol (Figure 5A). The binding stability was further analyzed by Gromacs2021.2 software through measurement of RMSD value, of which 0.1–0.3 nm indicated a relatively stable binding of the complex [35], and the hydrogen bonds numbers. The RMSD value of Vi-chloDHODH complex tended to be stable after 23 ns and stayed lower than 0.3 nm during the 100 ns (Figure 5B), and a generally 3–4 hydrogen bonds were formed between Vi and chloDHODH (Figure 5C), indicating a stable interaction between Vi and chloDHODH.

**Table 3:** **Proteins targeted by vidofludimus (only the top 10 proteins are listed).**

Target	Common name	Uniprot ID	ChEMBL ID	Target class	Probability*
Dihydroorotate dehydrogenase	DHODH	Q02127	CHEMBL1966	Oxidoreductase	1.000
Epidermal growth factor receptor erbB1	EGFR	P00533	CHEMBL203	Kinase	0.109
Fibroblast growth factor receptor 1	FGFR1	P11362	CHEMBL3650	Kinase	0.109
Peroxisome proliferator-activated receptor gamma	PPARG	P37231	CHEMBL235	Nuclear receptor	0.109
Aldose reductase (by homology)	AKR1B1	P15121	CHEMBL1900	Enzyme	0.109
Liver glycogen phosphorylase	PYGL	P06737	CHEMBL2568	Enzyme	0.109
Muscle glycogen phosphorylase	PYGM	P11217	CHEMBL3526	Enzyme	0.109
Peroxisome proliferator-activated receptor alpha	PPARA	Q07869	CHEMBL239	Nuclear receptor	0.109
Matrix metalloproteinase 12	MMP12	P39900	CHEMBL4393	Protease	0.109
ADAMTS5	ADAMTS5	Q9UNA0	CHEMBL2285	Protease	0.109

### Vidofludimus inhibited PRRSV replication through suppression of UMP synthesis in host cells

**Figure 6:**
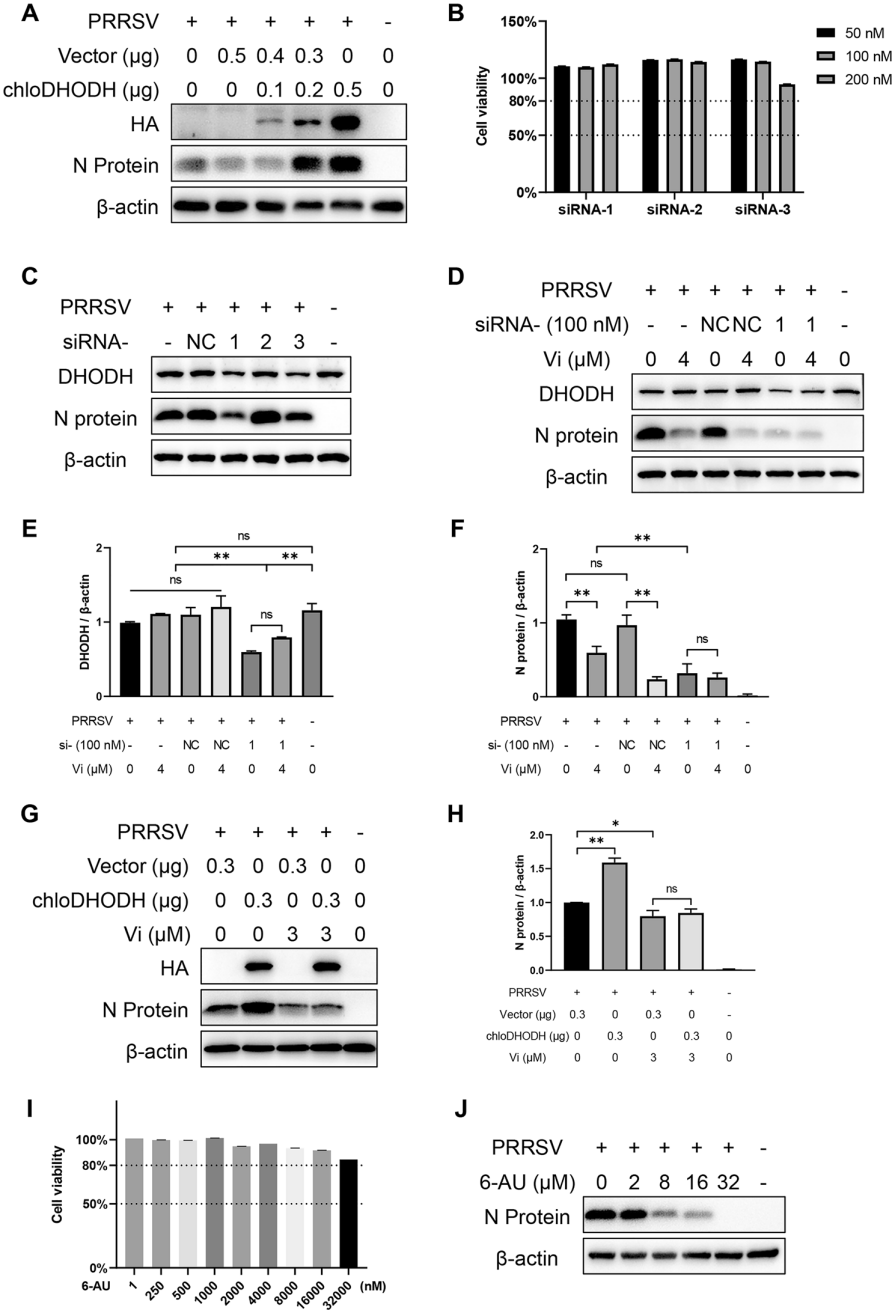
**Vidofludimus anti-PRRSV activity is mediated by DHODH. A** Effect of DHODH overexpression on PRRSV replication in Marc-145 cells. **B** Viability of Marc-145 cells treated with the indicated concentrations of siRNAs for 48 h. **C** Effect of DHODH interference on PRRSV replication in Marc-145 cells. **D** Effect of DHODH interference on Vi anti-PRRSV activity in Marc-145 cells. **E** and** F** Image J analysis of DHODH and PRRSV N protein quantification in (D). **G** Effect of Vi treatment on DHODH proviral activity in Marc-145 cells. **H** Image J analysis of PRRSV N protein quantification in (G). **I** Viability of Marc-145 cells treated with the indicated concentrations of 6-AU for 48 h. **J** Western blot of N-protein in PRRSV-infected cells with indicated concentrations of 6-AU or DMSO.

As DHODH was possibly a host target of Vi, we next investigated the role of DHODH during PRRSV replication. The overexpression of chloDHODH in Marc-145 cells showed a dose-dependent promotion activity on PRRSV replication (Figure 6A), and knockdown of chloDHODH gene by siRNA-1/3 restrained PRRSV replication (Figures 6B, C). When Vi was added into the culture medium, significant reduction of PRRSV N protein levels only occurred in the non-interference and si-NC groups but not in si-DHODH group (Figures 6D–F), indicating that the antiviral activity of Vi was achieved by targeting DHODH, which is consistent with the results of computer simulation analysis. Meanwhile, as shown in Figures 6G, H, Vi treatment reversed the effect of overexpressed DHODH on PRRSV replication. 6-azauracil (6-AU) is a potent inhibitor of Orotidine 5’-monophosphate decarboxylase (ODCase), which serves as a downstream enzyme of DHODH, catalyzing orotidine 5'-monophosphate (OMP) to uridine 5'-monophosphate (UMP). Further investigation showed that 6-AU could also inhibited PRRSV replication in a dose-dependent manner (Figures 6I, J), giving a hint that DHODH affected PRRSV replication through its activity in UMP synthesis.

**Figure 7:**
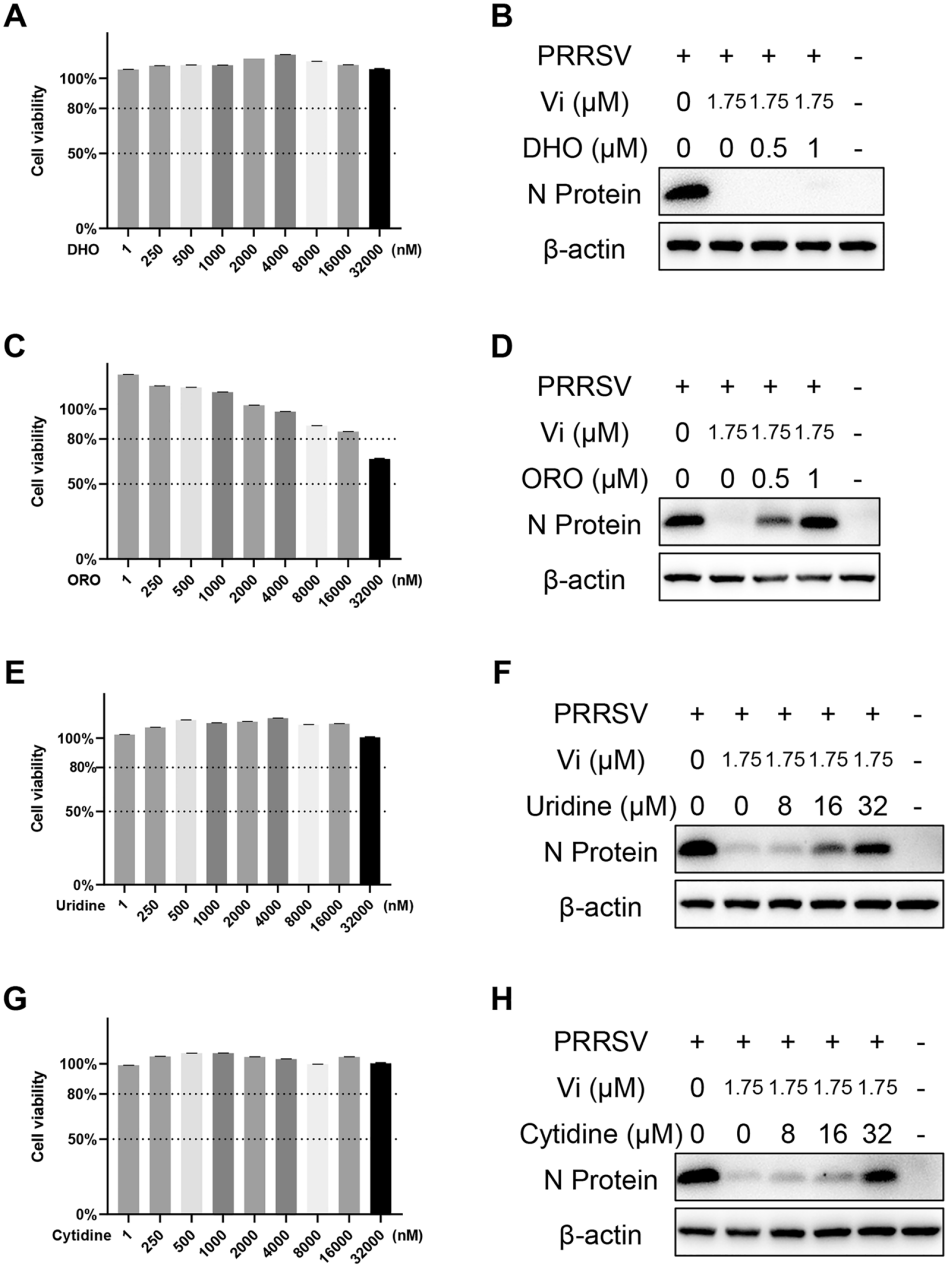
**Effect of DHO, ORO, uridine, and cytidine on vidofludimus anti-PRRSV activity.** Marc-145 cells were infected with PRRSV (0.1 MOI) with treatment of Vi and indicated concentrations of DHO, ORO, uridine, or cytidine for 48 h. DMSO served as the treatment control. **A, C, E,** and** G** Viability of Marc-145 cells treated with the indicated concentrations of DHO, ORO, uridine, or cytidine. **B, D, F,** and** H** Western blot of N-protein in cells infected with PRRSV and treated with the indicated compounds or DMSO.

DHODH is an oxidoreductase catalyzing dihydroorotate to orotate for UMP synthesis [36]. To further verify if chloDHODH affects PRRSV replication through its activity in UMP synthesis, a series of host molecules in UMP synthesis pathway were detected for their effect in Vi-PRRSV interaction. As shown in Figure 7, addition of dihydroorotate (DHO) did not reverse the inhibition activity of Vi on PRRSV replication. However, addition of orotate (ORO), uridine, and cytidine broke the anti-PRRSV activity of Vi in a dose-dependent manner. These results suggested that Vi inhibited PRRSV replication through the UMP synthesis pathway by interaction with DHODH.

### Vidofludimus has broad-spectrum antiviral activity against other swine disease viruses

**Figure 8:**
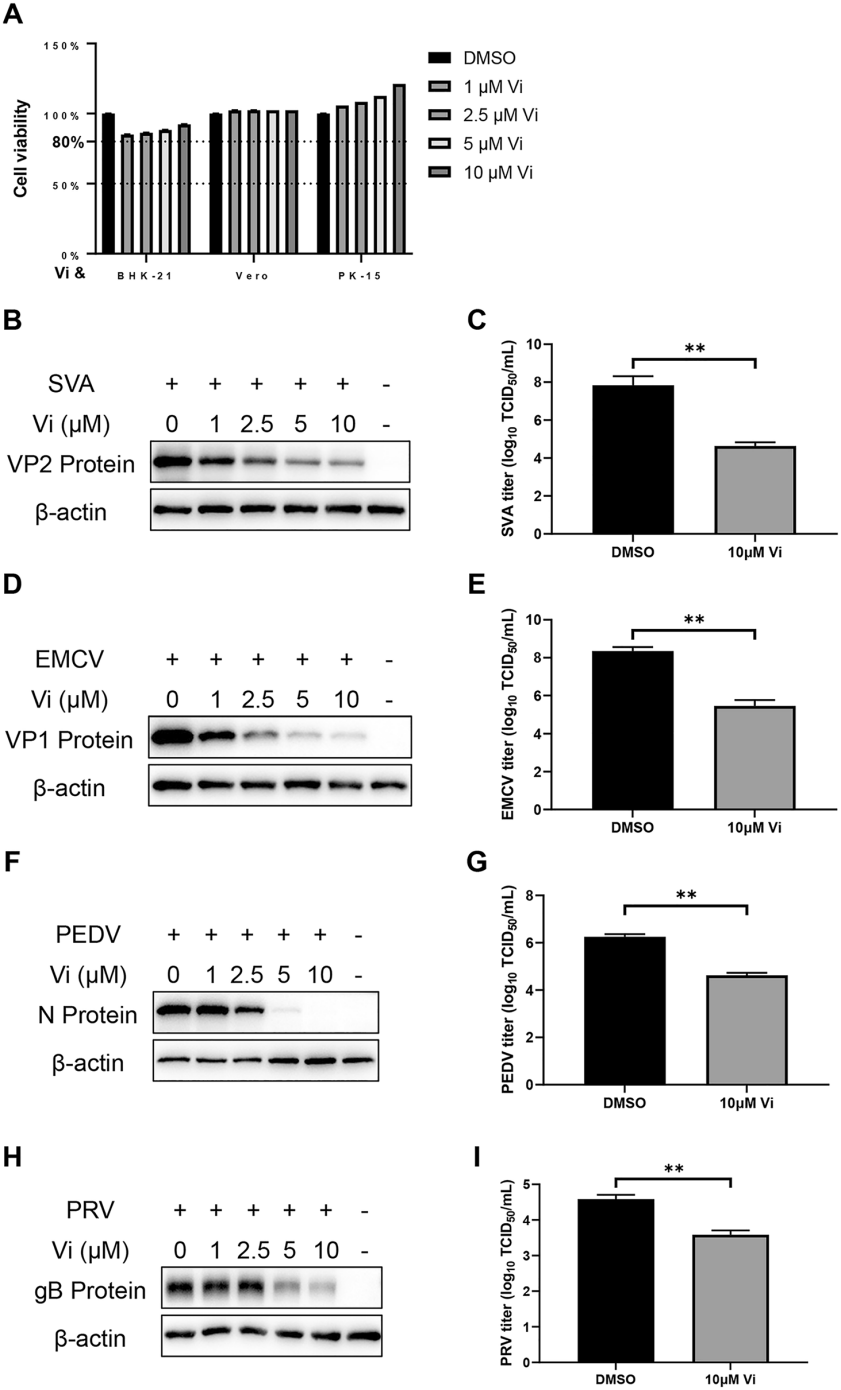
**Broad-spectrum antiviral activity of vidofludimus against SVA, EMCV, PEDV, and PRV. A** Viability of BHK-21, Vero, and PK-15 cells pretreated with the indicated concentrations of Vi for 18 h, 16 h, and 20 h, respectively. Western blot and TCID_50_ were used to examine the inhibition activity of Vi against SVA (**B** and** C**), EMCV (**D** and **E**), PEDV **F** and **G** and PRV (**H** and** I**). Error bars represent the mean ± SD. *, *P* < 0.05; **, *P* < 0.01; ***, *P* < 0.001; ****, *P* < 0.0001; ns: not significant.

The antiviral activity of Vi was further investigated with Seneca valley virus (SVA), encephalomyocarditis virus (EMCV), porcine epidemic diarrhea virus (PEDV) and pseudorabies virus (PRV). As shown in Figure 8, Vi exhibited a dose-dependent antiviral activity against SVA, EMCV, PEDV, and PRV within the safe concentration range. Structural analysis of ratDHODH in BHK-21 cells, chloDHODH in Vero cells, and susDHODH in PK-15 cells showed a highly coincident structure among them (Additional files 2A and B), and the binding between Vi and ratDHODH/susDHODH showed a binding energy of -9.23 and -8.32 kcal/mol, respectively (Additional file 2C), indicating that the antiviral activity of Vi against SVA, EMCV, PEDV and PRV was also associated with the DHODH-mediated pyrimidine metabolism.

## Discussion

PRRSV is one of the most important causative agents in swine production worldwide and causes huge economic losses every year [37]. Unfortunately, the current vaccines and immunization strategies cannot be effectively protective due to the high diversity of PRRSV strains, Therefore, novel strategies with a broad spectrum of protectiveness are urgently in need. In this study, an FDA-approved drug library was screened and four-hit compounds were firstly identified with anti-PRRSV activity from 2339 compounds. Among them, Vi exhibited a most substantial antiviral effect with the highest select index of 24.01.

Vi is a multifunctional molecule with immunoregulation and anti-inflammatory activity, being a potential treatment option for SARS-CoV-2 [38–40]. Here the anti-PRRSV activity of Vi was identified and the mechanisms were further explored. Vi showed an antiviral activity against different genotypes of PRRSV with little cytotoxicity to host cells. A time-of-addition analysis showed that Vi inhibited PRRSV infection in virus replication stage. Vi was predicted as a conjugate of DHODH, which is a key rate-limiting enzyme during the de novo synthesis of pyrimidine. Further exploration found that DHODH was an important promoter for PRRSV infection as overexpression of DHODH significantly promoted PRRSV replication while knockdown of DHODH significantly inhibited PRRSV replication. Moreover, knockdown of DHODH eliminated the antiviral activity of Vi against PRRSV, indicating that Vi might suppress PRRSV replication through DHODH. As 6-AU, a potent inhibitor of ODCase, inhibited PRRSV replication in a dose-dependent manner, it was suspected that Vi-DHODH effect on PRRSV replication could also be associated with the UMP synthesis. Addition of a series of host molecules in UMP synthesis pathway during Vi treatment on PRRSV infection confirmed that the anti-PRRSV activity of Vi was accomplished by suppressing UMP synthesis through blocking the oxidoreductase catalyzing activity of DHODH. As UMP synthesis is an important intermediate link to pyrimidine synthesis in mitochondria, it is believed that the host pyrimidine synthesis should be vital for PRRSV replication. As early as 2010, Kulkarni et al. found that 4SC-101 (synonym of Vi) significantly inhibits DHODH enzyme activity in humans, rats, and mice [41]. Subsequently, multiple research teams independently reported that DHODH inhibitor Vi has therapeutic effects on various diseases, including systemic lupus erythematosus, inflammatory bowel disease, renal transplantation rejection reaction, and relapsing–remitting multiple sclerosis [38, 39, 41, 42]. In recent years, there have been reported that DHODH inhibitors IMU-838 (synonym of Vi) and teriflunomide could antagonize viral infections such as SARS-CoV-2 and West Nile virus, respectively [40, 43]. In order to confirm the Vi treatment affecting pyrimidine biosynthesis, we used the *E. coli* expression system to express chloDHODH for conducting a biochemical experiment. However, we could not obtain the chloDHODH protein with enzymatic activity. This experiment should be done to confirm the Vi treatment affecting pyrimidine biosynthesis in the future.

DHODH is a general antiviral target as some antivirals with broad spectrums against negative-sense RNA viruses, positive-sense RNA viruses, DNA viruses, retroviruses, flaviviruses, cytomegaloviruses, adenoviruses, and coronaviruses have also been reported to play a DHODH-mediated antiviral activity [44–47]. This gives a hint that the antiviral activity of Vi might also be broad-spectrum. Further research in this study confirmed this as Vi exhibited a dose-dependent antiviral activity against SVA, EMCV, PEDV, and PRV in different cell types originated from various species. We next analyzed the structures of DHODH proteins from different species, and the binding activities between them and Vi. It showed that the DHODH proteins of *rat*, *sus scrofa*, and *chlorocebus sabaeus* showed a highly conserved structure and a strong interaction possibility with Vi (Additional file 2 and Figure 5). These results suggested that Vi could be an effective antiviral against various viruses whose replication rely on the DHODH-mediated host pyrimidine synthesis.

Our results also showed that Vi could inhibit PRRSV infection in virus binding stage. As heparan sulfate proteoglycan 2 (HSPG2), syndecan-4 (Sdc-4), CD163, and heparanase (HPSE) are closely included during PRRSV binding and entry [48–50], the mRNA synthesis of these four genes were detected during Vi suppression on PRRSV infection. The results showed that the mRNAs of HSPG2, Sdc-4, and CD163, but not HPSE, were significantly downregulated after Vi treatment (Additional file 3), indicating that HSPG2, Sdc-4, and CD163 might also be the targets of Vi for its antiviral activity. Further mechanisms are still under exploration.

**Figure 9:**
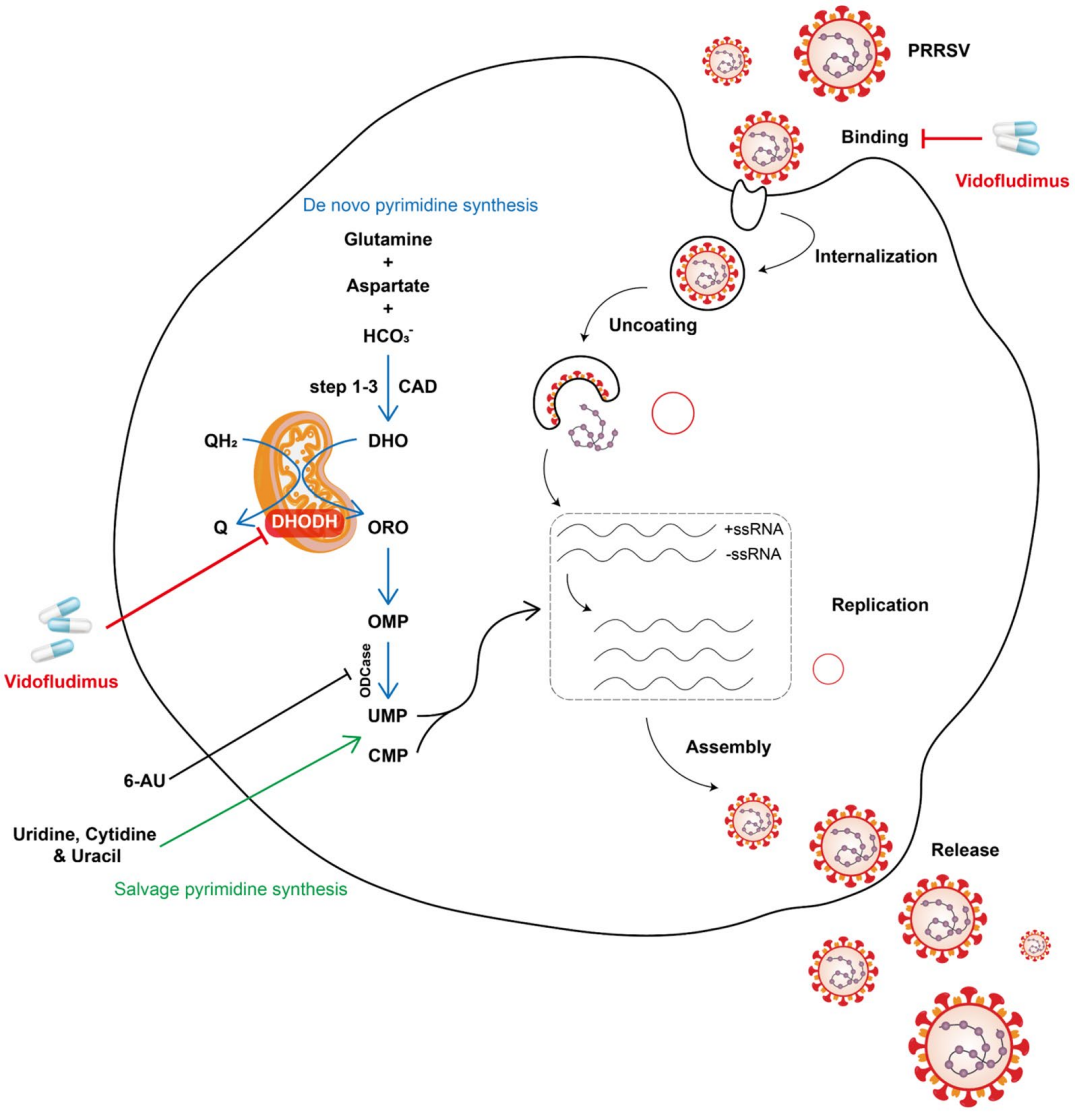
**The schematic diagram of PRRSV inhibition by vidofludimus.** Dihydroorotate dehydrogenase (DHODH), the key rate-limiting enzyme of the fourth step reaction in the de novo synthesis of pyrimidine (blue arrow), is important in generating UMP required for viral replication. By targeting and inhibiting DHODH activity, vidofludimus (Vi) blocks the production of orotate (ORO) and finally inhibits the synthesis of viral RNA. In addition, the effect of DHODH inhibitors can be disturbed by the salvage pathway (green arrow). On the other hand, Vi can also inhibit PRRSV adsorption on target cells, and its mechanism may be related to PRRSV adsorption-related protein receptors.

As concluded in Figure 9, Vi, a potent DHODH inhibitor, effectively inhibited PRRSV and other swine viruses’ infection by blocking the de novo pyrimidine biosynthesis pathway. These findings provide a new and promising therapeutic possibility of Vi for combating infections caused by pathogens in need of host pyrimidines. Future research should focus on the assessment and improvement of the antiviral activity of DHODH inhibitors in susceptible hosts as well as their security and efficacies in vivo in combination with vaccines for disease control.

## Supplementary Information

**Additional file 1: Validation of the 3D structure of *chlorocebus sabaeus* DHODH (chloDHODH). A** The Ramachandran plot statistics represent the most favorable, additional allowed, generously allowed, and disallowed region with a percentage of 94.1, 5.9, 0, and 0%, respectively. **B** Z-score of chloDHODH is -9.57.

**Additional file 2: Structure analysis of DHODHs and docking. A** Validation of the 3D structure of *sus scrofa* DHODH (susDHODH). **B** Comparative analysis of *rat* DHODH (ratDHODH, PDB: 1UUO), chloDHODH, and susDHODH structures by PyMOL. The structures of ratDHODH, chloDHODH, and susDHODH are labeled cyan, green, and purple, respectively. **C** Docked conformations of Vi with ratDHODH and susDHODH. The compound Vi is colored orange. The protein ratDHODH is colored cyan, susDHODH is colored purple, and the binding sites are shown as cavity structures. The binding energy of the Vi-ratDHODH or Vi-susDHODH complex, calculated using Autodock, is marked with an asterisk.

**Additional file 3: Vidofludimus effect on HSPG2, Sdc-4, CD163 and HPSE mRNA production.** Marc-145 cells were treated with 10 µM Vi for 4, 8, 16, and 24 h, followed by qRT-PCR for HPSE (**A**), HSPG2 (**B**), Sdc-4 (**C**), and CD163 (**D**) mRNA levels. The results are from one of three independent experiments. Error bars represent the mean ± SD. *, *P* < 0.05; **, *P* < 0.01; ***, *P* < 0.001; ****, *P* < 0.0001; ns: not significant.

## Data Availability

The data and materials will be made available on reasonable request.

## Abbreviations

OMP: orotidylic acid
UMP: uridine monophosphate
UDP: uridine diphosphate
UTP: uridine triphosphate
CTP: cytidine triphosphate
CDP: cytidine diphosphate
CMP: cytidine monophosphate
CAD: complex of the following three enzymes: carbamoyl-phosphate synthetase 2, aspartate carbamoyl transferase and dihydroorotase
DHO: dihydroorotate
DHODH: dihydroorotate dehydrogenase
UMPS: uridine monophosphate synthetase
ORO: orotate
QH_2_: ubiquinol
Q: ubiquinone
ODCase: orotidine 5'-phosphate decarboxylase
6-AU: 6-azauracil
CMPK: cytidine/uridine monophosphate kinase
NDPK: nucleoside diphosphate kinase
CTPS: cytidine triphosphate synthetase
UPP 1/2: uracil phosphoribosyltransferase 1 and uracil phosphoribosyltransferase 2
CDA: cytidine deaminase
UCK 1/2: uridine-cytidine kinase 1 and uridine-cytidine kinase 2
UPRT: uracil phosphoribosyl transferase
