# Correction: Vidofludimus inhibits porcine reproductive and respiratory syndrome virus infection by targeting dihydroorotate dehydrogenase

**DOI:** 10.1186/s13567-024-01447-y

**Published:** 2024-12-27

**Authors:** Yuanqi Yang, Yanni Gao, Lujie Zhang, Xing Liu, Yangyang Sun, Juan Bai, Ping Jiang

**Affiliations:** 1 Key Laboratory of Animal Diseases Diagnostic and Immunology, Ministry of Agriculture, MOE International Joint Collaborative Research Laboratory for Animal Health & Food Safety, College of Veterinary Medicine, Nanjing Agricultural University, Nanjing, 210095 China; 2 Jiangsu Co-Innovation Center for the Prevention and Control of Important Animal Infectious Diseases and Zoonoses, Yangzhou University, Yangzhou, 225009 China

**Correction: Veterinary Research (2023) 54:124** 10.1186/s13567-023-01251-0

Following publication of the original article [1], the authors identified an error in the Funding section.

The incorrect Funding is:

This work was supported by the National Natural Science Foundation (32230103), the Earmarked Fund for CARS-35, the Jiangsu Independent Innovation Fund Project (CX (22) 1006), and the Priority Academic Program Development of Jiangsu Higher Education Institutions (PAPD). The funders had no role in study design, data collection and analysis, decision to publish, or preparation of the manuscript.

The correct Funding is:

This work was supported by the National Natural Science Foundation (32230103), the Earmarked Fund for CARS-35, the Jiangsu Independent Innovation Fund Project (CX (22) 1011), and the Priority Academic Program Development of Jiangsu Higher Education Institutions (PAPD). The funders had no role in study design, data collection and analysis, decision to publish, or preparation of the manuscript.
