# Supplementary figures and images for: Vidofludimus inhibits porcine reproductive and respiratory syndrome virus infection by targeting dihydroorotate dehydrogenase
Source: Vet Res. 2023 Dec 20;54:124. doi: 10.1186/s13567-023-01251-0 (PMC10731701; doi:10.1186/s13567-023-01251-0)

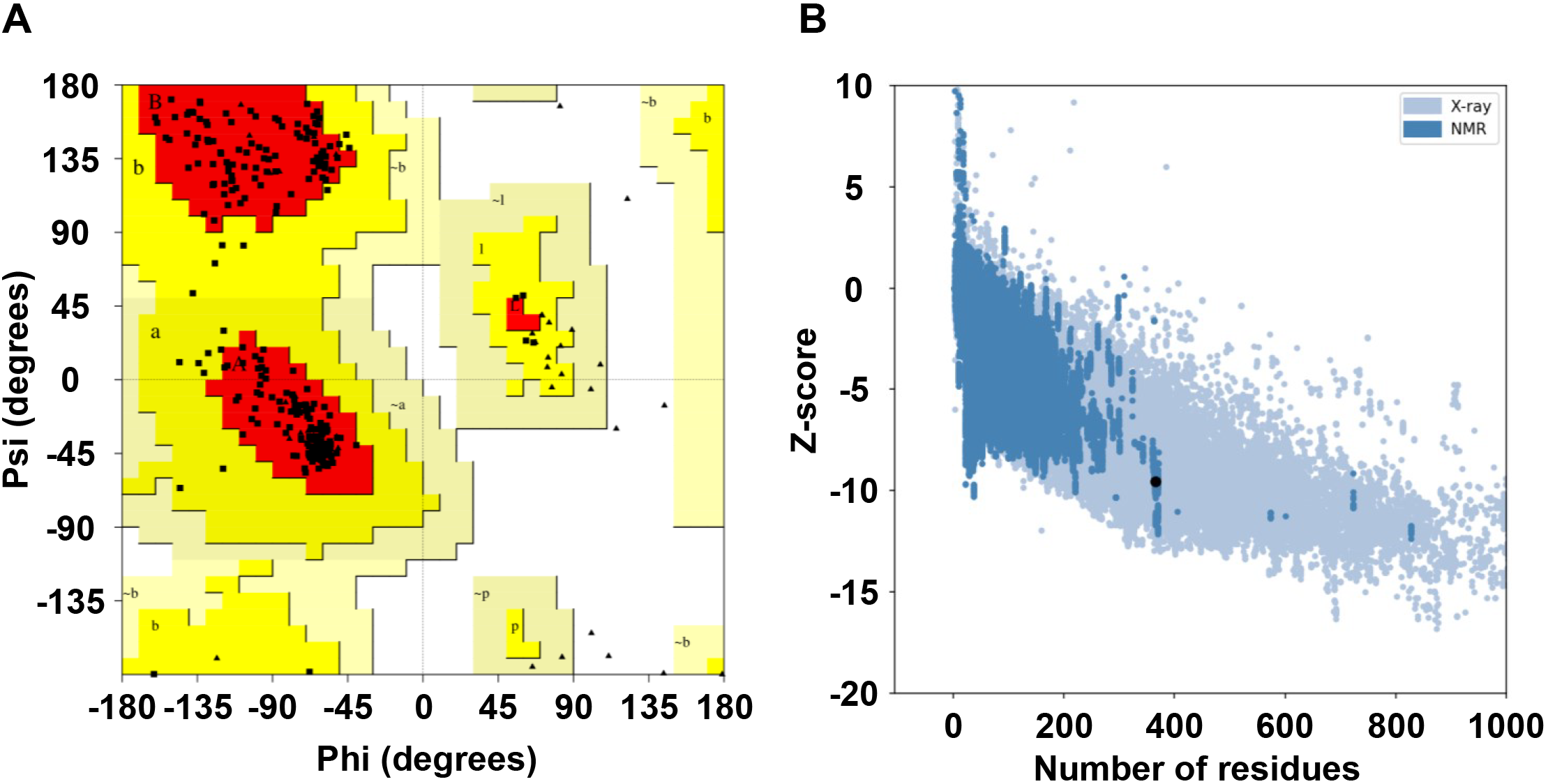

Supplement: Supplementary file 1 — Additional file 1: Validation of the 3D structure of chlorocebus sabaeus DHODH (chloDHODH). A The Ramachandran plot statistics represent the most favorable, additional allowed, generously allowed, and disallowed region with a percentage of 94.1, 5.9, 0, and 0%, respectively. B Z-score of chloDHODH is -9.57. [file 13567_2023_1251_MOESM1_ESM.tif]

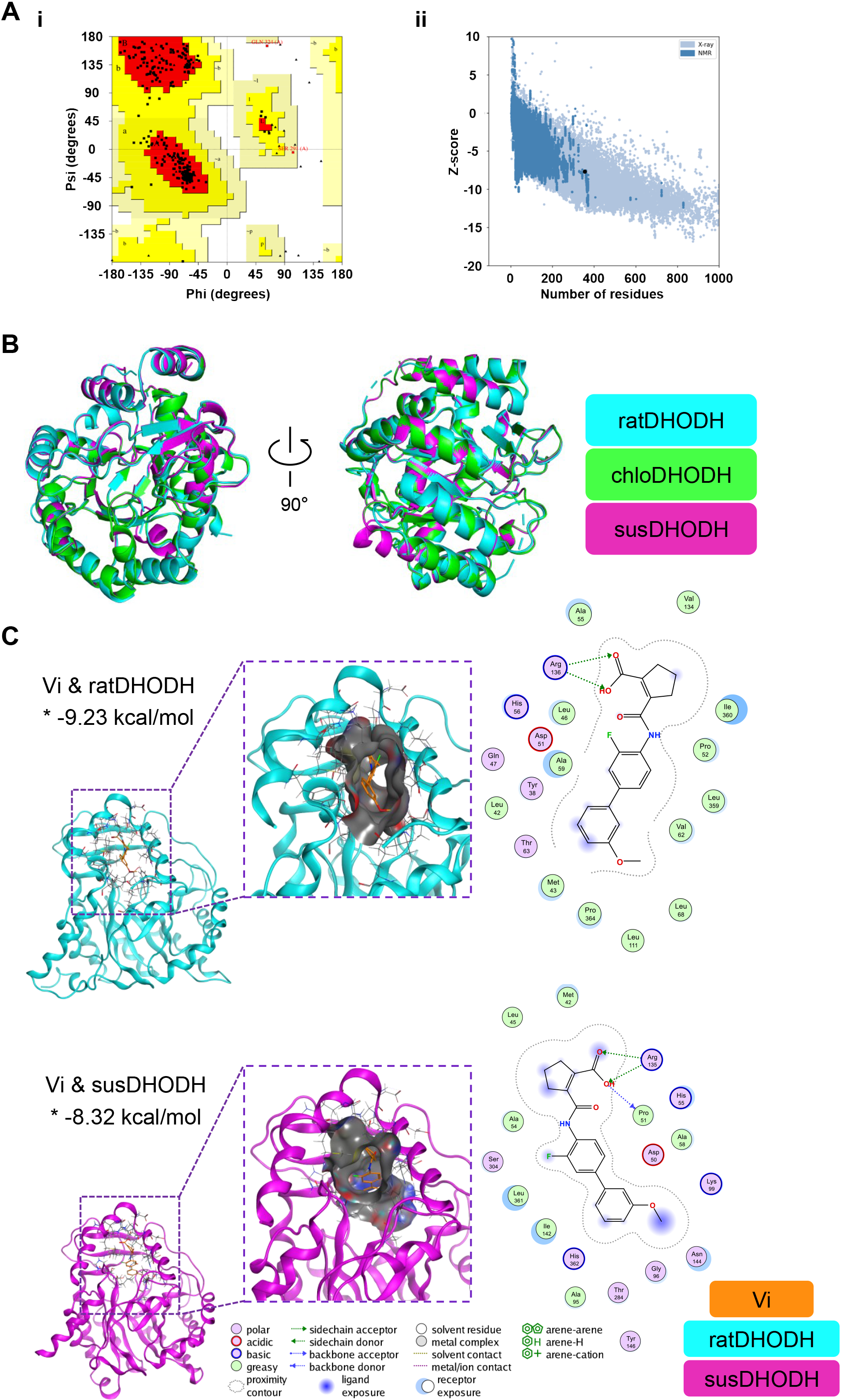

Supplement: Supplementary file 2 — Additional file 2: Structure analysis of DHODHs and docking. A Validation of the 3D structure of sus scrofa DHODH (susDHODH). B Comparative analysis of rat DHODH (ratDHODH, PDB: 1UUO), chloDHODH, and susDHODH structures by PyMOL. The structures of ratDHODH, chloDHODH, and susDHODH are labeled cyan, green, and purple, respectively. C Docked conformations of Vi with ratDHODH and susDHODH. The compound Vi is colored orange. The protein ratDHODH is colored cyan, susDHODH is colored purple, and the binding sites are shown as cavity structures. The binding energy of the Vi-ratDHODH or Vi-susDHODH complex, calculated using Autodock, is marked with an asterisk. [file 13567_2023_1251_MOESM2_ESM.tif]

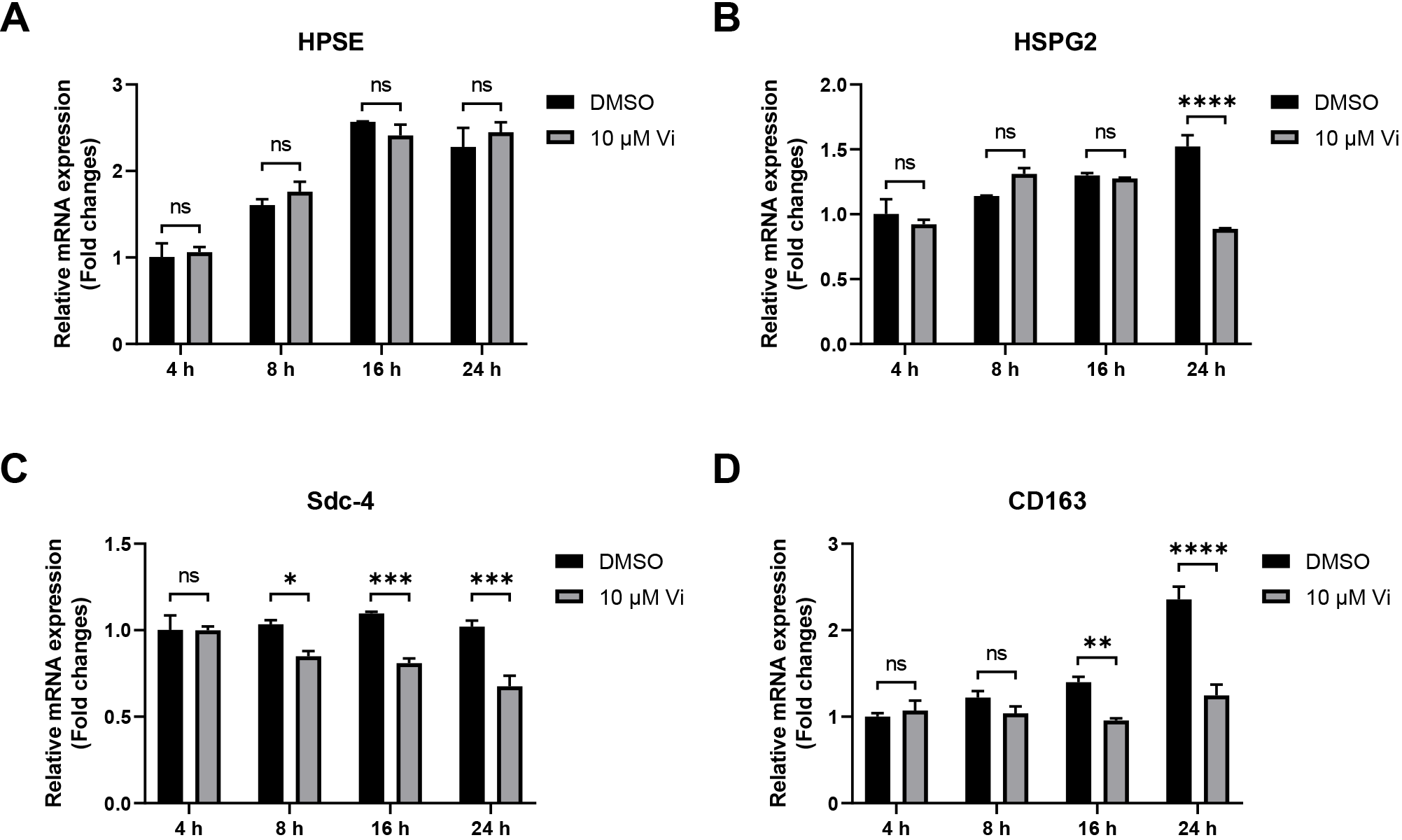

Supplement: Supplementary file 3 — Additional file 3: Vidofludimus effect on HSPG2, Sdc-4, CD163 and HPSE mRNA production. Marc-145 cells were treated with 10 µM Vi for 4, 8, 16, and 24 h, followed by qRT-PCR for HPSE (A), HSPG2 (B), Sdc-4 (C), and CD163 (D) mRNA levels. The results are from one of three independent experiments. Error bars represent the mean ± SD. *, P < 0.05; **, P < 0.01; ***, P < 0.001; ****, P < 0.0001; ns: not significant. [file 13567_2023_1251_MOESM3_ESM.tif]
